# Supplementary material for: A Novel ALDH2 Activator AD-9308 Improves Diastolic and Systolic Myocardial Functions in Streptozotocin-Induced Diabetic Mice
Source: Antioxidants (Basel). 2021 Mar 13;10(3):450. doi: 10.3390/antiox10030450 (PMC7998151; doi:10.3390/antiox10030450)
Supplement: Supplementary file 1 [file antioxidants-10-00450-s001.pdf]

antioxidants-1113200-Supplementary

1. Materials and Methods

Measurement of cardiomyocyte size

Cardiac tissue sections were subjected to hematoxylin and eosin (H&E) stain and were taken images using Olympus BX51 microscope. Two random fields from each of the one non-serial tissue sections per animal were analyzed. The quantification of myocyte diameter was determined using Image J software as described previously [1].

2. Figures

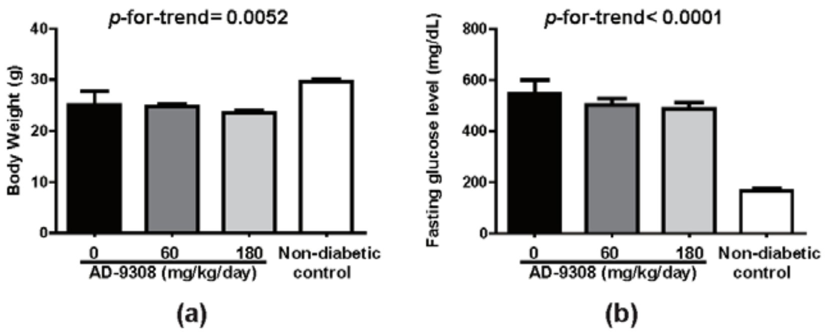

Figure S1. Characterization of the streptozotocin (STZ)-induced diabetic mice with AD-9308 treatment. The mean (**a**) body weight and (**b**) fasting blood glucose level were measured in STZ-induced diabetic mice treated with 0, 60, 180 mg/kg/day of AD-9308 by oral gavages and non-diabetic control mice at the age of 5 month. Data are presented as mean± SEM (n= 14-16). *p*-for-trend was used to test linear trend.

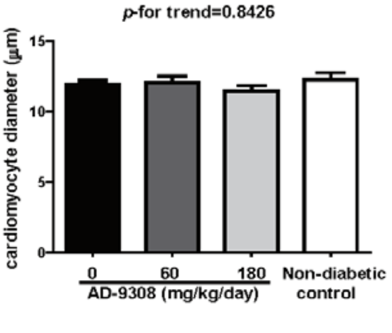

Figure S2. Cardiomyocyte size of the streptozotocin (STZ)-induced diabetic mice with AD-9308 treatment. The mean cardiomyocyte diameters were measured in cardiac tissue from STZ-induced diabetic mice treated with 0, 60, 180 mg/kg/day of AD-9308 by oral gavages and non-diabetic control mice. Data are presented as mean± SEM (n= 14-16). *p*-for-trend was used to test linear trend.

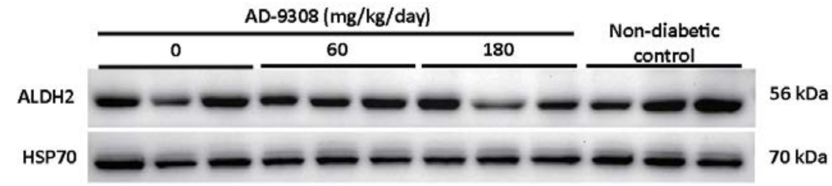

Figure S3. ALDH2 protein expression levels of the streptozotocin (STZ)-induced diabetic mice with AD-9308 treatment. Immunoblottings of ALDH2 were detected in cardiac tissue from STZ-induced diabetic mice treated with 0, 60, 180 mg/kg/day of AD-9308 by oral gavages and non-diabetic control mice.

3. Tables

Table S1. Primary antibodies used in this study.

Antibody	Concentration	Brand
4-HNE	1:1000	Sigma-Aldrich; 393207
HSP70	1:5000	Abcam; ab45133
Ho-1	1:2000	GeneTex; GTX101147
iNOS	1:2000	Cell Signaling Technology; 2982S
Bcl-2	1:2000	GeneTex; GTX100064
Bax	1:2000	GeneTex; GTX109683
Casp3	1:2000	GeneTex; GTX110543
GAPDH	1:5000	GeneTex; GTX100118
Iκbα	1:1000	GeneTex; GTX110521
p65	1:5000	Abcam; ab32536
α-tubulin	1:5000	Proteintech; HRP-60031
Histone H3	1:5000	Abcam; ab1791
Opa1	1:1000	GeneTex; GTX129917
Drp1	1:2000	Cell Signaling Technology; D6C7
Beclin 1	1:2000	GeneTex; GTX133555
Lc3A	1:2000	GeneTex; GTX132889
Lc3B	1:2000	GeneTex; GTX127375
Serca2	1:2000	ABclonal; A1097
Pln	1:2000	ABclonal; A17964

Table S2. RT-qPCR primer sequences used in this study.

primer	Gene ID	Forward primer	Reverse primer
18S	19791	ACGATGCCGACTGGCGATGC	TCCTGGTGGTGCCCTTCCGT
Tgf-β1	21083	CTCCCGTGGCTTCTAGTGC	GCCTTAGTTTGGACAGGATCTG
Ctgf	14219	AGAACTGTGTACGGAGCGTG	GTGCACCATCTTTGGCAGTG
Fsp1	20198	TGTAATTGTGTCCACCTTCC	GCTCATCACCTTCTGGAATG
Postn	50706	AAGTTGGCCTTAGCGACCTC	GCAGGGATTTCTCTGCTGGT
Fn-1	14268	TGGTGGCCACTAAATACGAA	GGAGGGCTAACATTCTCCAG
Tgf-β2	21808	CAGTGGGAAGACCCCACATC	TGTAAAGAGGGCGAAGGCAG
α-Sma	11475	GTACCCAGGCATTGCTGACA	GAGGCGCTGATCCACAAAAC
ColIV	12826	GGCAGACTCGGACCACTATG	TGGTACGTGTGGTAACTTCTCT

Il-1β	16176	CACAGCAGCACATCAACAAG	GTGCTCATGTCCTCATCCTG
Il-6	16193	TAGTCCTTCCTACCCCAATTTCC	TTGGTCCTTAGCCACTCCTTC
Infγ	15978	CGGCACAGTCATTGAAAGCCTA	GTTGCTGATGGCCTGATTGTC
Mcp-1	20296	GCTACAAGAGGATCACCAGCAG	GTCTGGACCCATTCCTTCTTGG
Sap	20219	TTTGGGTCAATGGAAAGCCTTGGG	TGACCTTTGAAACCCTCCTCCGTA
Tnf-α	21926	TACTGAACTTCGGGGTGATTGGTCC	CAGCCTTGTCCCTTGAAGAGAACC

Reference:

1. Kawai, K., Qin, F., Shite, J., Mao, W., Fukuoka, S., Liang, C.S. Importance of antioxidant and antiapoptotic effects of beta-receptor blockers in heart failure therapy. Am. J. Physiol. Heart Circ. Physiol. 2004, 287 (3): pp. H1003-H1012. [doi: 10.1152/ajpheart.00797.2003]
